# Encapsulated Indigenous Lactic Acid Bacteria Strains From Traditional Iranian Cheese Alleviate Hyperglycemia and Inflammation in Streptozotocin‐Induced Diabetic Rats

**DOI:** 10.1002/fsn3.71295

**Published:** 2025-12-06

**Authors:** Yousef Nami, Behnam Kafil, Alireza Dehnad

**Affiliations:** ^1^ Department of Food Biotechnology, Branch for Northwest & West Region, Agricultural Biotechnology Research Institute of Iran, Agricultural Research, Education and Extension Organization (AREEO) Tabriz Iran; ^2^ Department of Livestock Bacterial Diseases Research Razi Vaccine and Serum Research Institute, Agricultural Research, Education and Extension Organization (AREEO) Karaj Iran

**Keywords:** gut microbiota, inflammatory cytokines, insulin resistance, probiotics, type 2 diabetes mellitus

## Abstract

The current study was conducted to investigate the antidiabetic and anti‐inflammatory effectiveness of encapsulated indigenous lactic acid bacteria originating from traditional Iranian cheese. Two out of 40 gram‐positive, catalase‐negative strains were selected and identified as *Lactiplantibacillus pentosus* and *Lactiplantibacillus plantarum*. Both strains exhibited strong acid (pH 2.5) and bile (0.3%) tolerance, with survival rates exceeding 64%. In vitro hydrophobicity (> 63%), autoaggregation (> 66%), and coaggregation with *Escherichia coli* (over 51%) were observed. These strains also demonstrated the highest antimicrobial activity (inhibition zones up to 27 mm) and were selected for in vivo testing. Male Wistar rats (*n* = 32) were randomly assigned to four groups: normal control, diabetic control (STZ, 35 mg/kg), normal + probiotics, and diabetic + probiotics (1 × 10^9^ CFU/day, orally). At the end of 8 weeks, diabetic rats receiving encapsulated probiotic strains showed significantly lower fasting blood glucose (200.8 ± 8.4 vs. 317.1 ± 10.7 mg/dL in diabetic controls, *p* < 0.01), higher serum insulin levels (12.3 ± 1.0 vs. 9.3 ± 0.9 μIU/mL, *p* < 0.01), and better body weight retention (245 vs. 215 g, *p* < 0.05). Proinflammatory cytokines IL‐1β, IL‐6, and TNF‐α were significantly reduced in probiotic‐treated diabetic rats compared to untreated diabetic controls (*p* < 0.01). Probiotic delivery was well tolerated in normoglycemic rats, with no adverse effects reported. Overall, these findings support the potential of microencapsulated *L. pentosus* D1 and *L. plantarum* D2 as safe and effective adjuncts for managing type 2 diabetes by modulating glycemic and inflammatory responses.

## Graphical abstract

Microencapsulated *Lactiplantibacillus pentosus* D1 and *L. plantarum* D2, isolated from traditional Iranian cheese, exhibited robust probiotic properties and high encapsulation efficiency. In streptozotocin‐induced diabetic rats, oral administration (1 × 10^9^ CFU/day, 8 weeks) significantly reduced fasting blood glucose (200.8 ± 8.4 vs. 317.1 ± 10.7 mg/dL), elevated serum insulin (12.3 ± 1.0 vs. 9.3 ± 0.9 μIU/mL), improved body weight retention, and markedly lowered proinflammatory cytokines (IL‐1β, IL‐6, TNF‐α; *p* < 0.01), demonstrating safe, pathology‐specific antidiabetic and anti‐inflammatory efficacy.

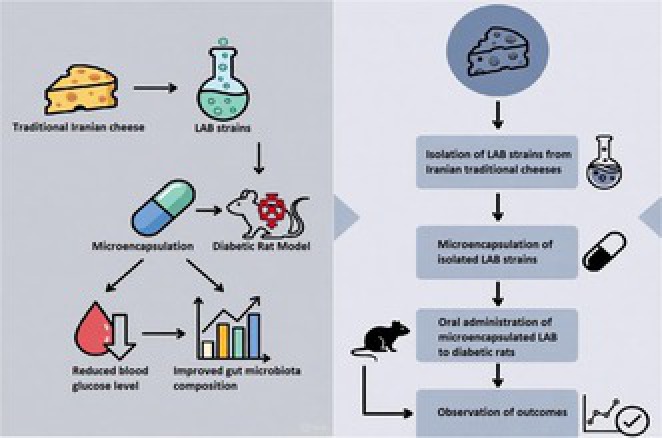

## 1 Introduction

With over 460 million individuals affected globally, type 2 diabetes mellitus (T2DM) represents one of the most prevalent and burdensome metabolic diseases worldwide. A recent systematic review concluded that the global burden of T2DM will continue to rise rapidly due to a perfect storm of accelerated lifestyle changes, increased rates of urbanization, deteriorating dietary patterns, and an aging population, with over 460 million people currently affected worldwide, with low‐ and middle‐income countries (LMICs) being disproportionately impacted (Samant et al. 2025). In addition to being a significant cause of mortality, T2DM is a major risk factor for cardiovascular diseases, kidney dysfunction, and neuropathies, which impose heavy economic burdens on healthcare systems (Giacco and Brownlee 2010). This suggests that consideration should be given to alternative microbiota‐based interventional strategies, such as the use of indigenous probiotic strains combined with encapsulation technologies. Pharmaceutical agents such as metformin and insulin remain the main treatment options for diabetes. However, their continued long‐term use can come with adverse side effects, including gastrointestinal distress, weight gain, and hypoglycemia, which have resulted in a search for safer and more natural forms of treatment (Subramaniam et al. 2021).

Recent research has increasingly focused on the gut microbiome and its role in metabolic health, highlighting the potential of probiotics in modulating gut flora to improve insulin sensitivity and glycemic control (Bock et al. 2024). Probiotics are live microorganisms that, when administered in adequate amounts, confer health benefits on the host as defined by the FAO/WHO expert consultation (Araya et al. 2002). Several strains of *Lactobacillus* and *Bifidobacterium* have been shown to improve lipid metabolism, reduce inflammatory cytokines, and modulate glucose homeostasis via strain‐specific mechanisms such as enhancing gut barrier integrity, promoting short chain fatty acid (SCFA) production, and modulating Toll‐like receptor pathways (Bagarolli et al. 2017; Yadav et al. 2013). Beyond glycemic control, probiotics have shown promise in attenuating chronic systemic inflammation, a central feature of T2DM pathophysiology, by modulating gut‐derived immune responses (Siavash et al. 2017; Subramaniam et al. 2021).

Despite promising outcomes, the clinical application of probiotics faces significant challenges, including strain viability, targeted delivery, and gastrointestinal survival (Burgain et al. 2011). Microencapsulation technologies have emerged as an effective strategy to enhance the stability and functional efficacy of probiotic formulations. These technologies protect probiotic cells from harsh gastric conditions, extend shelf life, and enable controlled release in the intestine (Nami et al. 2023; Rojas‐Muñoz et al. 2023). Such technologies are particularly relevant for delivering probiotics to diabetic individuals, whose altered gut environment may impair microbial colonization.

Traditional dairy products, such as artisanal cheeses, represent a valuable reservoir of indigenous probiotic strains with unique functional properties (Kouhi et al. 2022). Furthermore, innovative biosensor technologies, such as dual‐signal light detection using porous silicon Bragg mirrors for precise quantification of milk proteins like β‐lactoglobulin, highlight the growing intersection of advanced analytical methods with dairy‐derived probiotic sources, potentially aiding in strain selection and quality control for functional foods (Gao et al. 2022). In this context, our study aimed to isolate and identify native lactic acid bacteria (LAB) strains from traditional cheeses in northwestern Iran and evaluate their therapeutic potential in a rat model of T2DM. Using milk protein‐based microencapsulation and emulsification techniques, selected strains were administered orally to diabetic rats over an 8‐week intervention period. The study assessed the effects on fasting blood glucose, serum insulin levels, body weight, and proinflammatory cytokines (TNF‐α, IL‐1β, and IL‐6). To our knowledge, this is the first study to evaluate the antidiabetic and anti‐inflammatory effects of encapsulated LAB strains isolated from traditional Iranian cheeses.

## 2 Materials and Methods

### 2.1 Preparation of Cheese Samples for Bacterial Isolation

The selective culture medium used for isolating LAB species was de Man Rogosa and Sharpe (MRS), which is considered a specific medium capable of supporting the complex nutritional requirements of these bacteria. MRS agar and broth media were prepared according to the manufacturer's instructions using products supplied by Biolife (Italy) and Scharlau (Spain). For microbiological analysis, 5 g of the cheese sample was aseptically added to 45 mL of sterile 2% (w/v) sodium citrate solution, which had been preheated to 45°C. The mixture was homogenized for 1 min using a Shaker homogenizer. A portion of the resulting suspension was transferred via micropipette into 20 mL of sterile MRS broth. The culture was then incubated anaerobically at 37°C for 24 h to enrich bacterial populations. All steps were carried out under aseptic conditions inside a laminar flow hood (Haghshenas et al. 2023). This homogenization and enrichment step was employed to selectively promote LAB growth while minimizing contamination from non‐target microbiota, ensuring reliable isolation from complex cheese matrices.

### 2.2 Biochemical Identification Tests for LAB Strains

Fifteen traditional cheese samples were collected from local markets. From these, 40 bacterial isolates were obtained under aseptic conditions and subcultured on MRS agar plates at 37 C for 48 h under anaerobic conditions. Initial morphological and biochemical screening—including Gram staining, catalase testing, cell shape, and fermentative metabolism—identified 15 isolates as presumptive LAB.

#### 2.2.1 Gram Staining

Gram staining was performed following the manufacturer's protocol provided by Lab Tron (Wen et al. 2023), a commercial supplier of microbiological staining kits.

#### 2.2.2 Catalase Test

The catalase test was conducted by placing a small amount of bacterial culture on a glass slide and adding a drop of 3% hydrogen peroxide. The presence or absence of immediate bubble formation was recorded as the test result (MacFaddin 2000).

#### 2.2.3 Oxidase Test

The oxidase test was performed using the indirect filter paper method with Kovács reagent. A color change occurring within 5–10 s was considered a positive result (MacFaddin 2000).

#### 2.2.4 Citrate Utilization Test

Simmons' citrate agar was used to determine the ability of the isolates to utilize citrate as a sole carbon source. A color change in the medium was interpreted as a positive result, while no color change indicated a negative outcome (MacFaddin 2000).

#### 2.2.5 Indole, Motility, and Hydrogen Sulfide (H_2_S) Production

The indole production, bacterial motility, and H_2_S generation were assessed using a SIM (Sulfide‐Indole‐Motility) medium. The results for each parameter were recorded separately (MacFaddin 2000).

#### 2.2.6 Nitrate Reduction Test

Nitrate reduction was evaluated using McFaddin's standard protocol. The development of a red color after the addition of nitrate reagents or following the addition of zinc powder in cases where no initial color change occurred was used to interpret the results (MacFaddin 2000).

#### 2.2.7 Carbohydrate Fermentation Test

Carbohydrate fermentation was assessed using a phenol red broth base containing individual sugars. Color change in the medium was used as an indicator of acid production and positive fermentation (MacFaddin 2000).

### 2.3 Molecular Identification

The 16S rRNA gene (~1500 bp) of each isolate was amplified to the level of molecular identification using lactic acid bacteria (LAB)‐specific primers forward primer 5′‐GAG AGT TTG ATC CTG GCT CAG‐3′ and reverse primer 5′‐GAA AGG AGG GTG ATC CAG CC‐3′. The PCR amplifications were conducted in a thermal cycler with the following assumptions: initial denaturation at 95°C for 5 min, then amplification for 30 cycles at 94°C for 60 s (denaturing), before annealing at 53°C for 60 s, followed by extension for 120 s at 72°C, with a final extension step at 72°C for 5 min. Each amplicon was validated by agarose gel electrophoresis and purified before sequencing. DNA sequencing was performed by Macrogen (Seoul, South Korea), and the resulting sequences were aligned using Clustal W sequence alignment. To identify the closest phylogenetic associates of the obtained sequences, BLAST analysis was performed against the NCBI GenBank database to identify the closest phylogenetic relatives based on sequence similarity (Nami et al. 2018). BLAST analysis revealed 99.8% and 99.9% sequence identity for strains D1 and D2 to *Lactiplantibacillus pentosus* DSM 20186T and *Lactiplantibacillus plantarum* WCFS1, respectively. The sequences have been deposited in GenBank under accession numbers PV992300 (D1) and PV992304 (D2).

**FIGURE 1:**
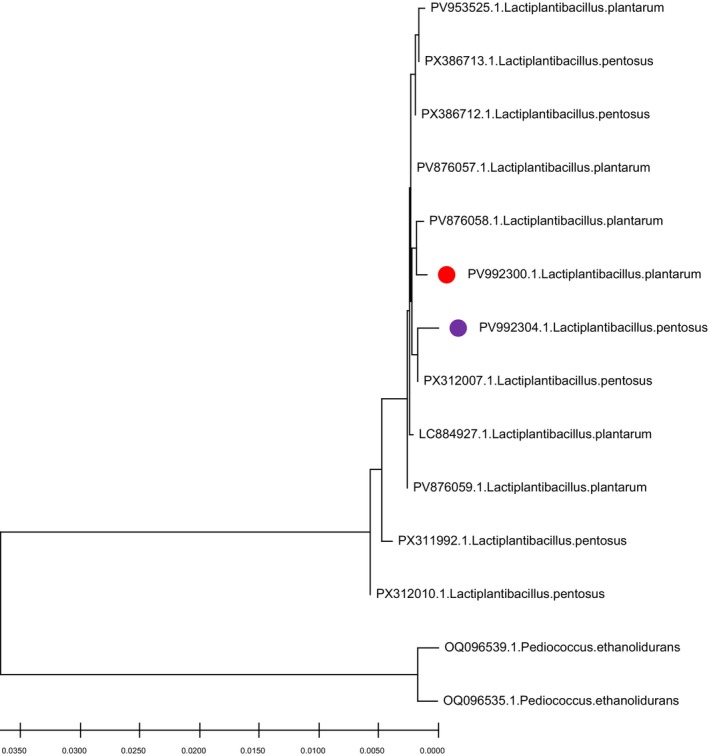
Phylogenetic tree constructed using the neighbor‐joining method in MEGA software (version 11) based on partial 16S rRNA gene sequences. Strains D1 and D2 cluster within the *Lactiplantibacillus pentosus* (bootstrap support: 98%) and *Lactiplantibacillus plantarum* (bootstrap support: 99%) clades, respectively. Bootstrap values (1000 replications) are indicated at nodes. Scale bar represents 0.001 substitutions per nucleotide position.

Phylogenetic analysis was conducted using the neighbor‐joining method in MEGA software (version 11), incorporating 1000 bootstrap replications to assess tree robustness. The partial 16S rRNA sequences of D1 and D2 were aligned with reference sequences from closely related strains, including *L. pentosus* (GenBank: PX386713), *L. pentosus* (GenBank: PX386712), *L. pentosus* (GenBank: PX312007), *L. pentosus* (GenBank: PX311992), *L. pentosus* (GenBank: PX312010), *L. plantarum* (GenBank: PV953525), *L. plantarum* (GenBank: PV876057), *L. plantarum* (GenBank: PV876058), *L. plantarum* (GenBank: PV884927), *L. plantarum* (GenBank: PV876059), and two outgroup species (e.g., *Pediococcus ethanolidurans* , GenBank: OQ096539, and *Pediococcus ethanolidurans* , GenBank: OQ096535). The resulting tree (Figure 1) positioned strain D1 within the *L. pentosus* clade (bootstrap support: 98%) and strain D2 within the *L. plantarum* clade (bootstrap support: 99%), confirming their distinct phylogenetic affiliations despite high 16S rRNA similarity (> 99%) between the species. This analysis aligns with prior studies demonstrating that 16S rRNA phylogeny, supplemented by recA or multi‐locus markers, effectively differentiates members of the *L. plantarum* group (Zheng et al. 2020). 16S rRNA gene sequencing was selected for its high resolution in differentiating closely related *Lactiplantibacillus* species, providing robust phylogenetic confirmation beyond biochemical tests.

### 2.4 In Vitro Probiotic Characterization

#### 2.4.1 Acid and Bile Salt Tolerance Assays

To initially screen the probiotic capacities of the isolates, we evaluated their tolerance to acidic and bile salt conditions, measuring any resulting changes in optical density under these stress conditions (Hernández‐Montoliu et al. 2023). Overnight cultures were prepared in aerated MRS broth (37°C, 24 h) and then centrifuged (3000 rpm, 10 min) prior to dissolving the bacterial pellet under the treatment conditions. Tolerance to acid was evaluated in the MRS broth adjusted for pH 2.5 for 3 h at 37 C. Tolerance to bile salts was determined by maintaining bacterial cells in MRS with 0.3% (w/v) oxgall (dehydrated bile; Becton and Dickinson), pH 6.8, for 4 h under mild agitation (Mazlumi et al. 2022). The optical density at 600 nm (OD_600_) was used to monitor the optical density at 600 nm (OD_600_) before and after the treatment. Each condition was assayed in triplicate. The survival of the bacteria was expressed in percentage using the formula:

$$\text{Survival}\;\left(\%\right)=\frac{\mathrm{OD}\;\text{after treatment}}{\;\text{before treatment}}\times 100$$

This assay enabled the selection of the most tolerant strains for subsequent evaluations based on their ability to survive simulated gastrointestinal conditions. These conditions (pH 2.5 for 3 h; 0.3% bile for 4 h) simulate gastric and small intestinal stress, respectively, to predict in vivo survival—a critical FAO/WHO criterion for probiotic functionality.

#### 2.4.2 Antimicrobial Activity

The antimicrobial activity of the selected potential probiotic isolates was assessed using the agar disc diffusion assay. Standard pathogenic bacteria, including *Staphylococcus aureus* (PTCC 1112), *Escherichia coli* (PTCC 1270), *Listeria monocytogenes* (PTCC 1295), and *Salmonella typhimurium* (PTCC 1735), were obtained from the Iranian Research Organization for Science and Technology (IROST). From the 24‐h culture of selected potential probiotic isolates prepared in MRS broth, about 4–5 uniform colonies were inoculated into fresh MRS broth, which was again incubated under microaerophilic conditions at 37 C for 4 days to allow for antimicrobial metabolite production. Tubes were sealed with sterile paraffin to ensure semi‐anaerobic conditions. After incubation, the paraffin was removed, and then the cultures were centrifuged at 3000×*g* for 10 min to obtain cell‐free supernatant (CFS). The supernatant was transferred to sterile tubes and filtered twice using a membrane filter of 0.2 μm pore size. To check for the presence of bacterial cells in the filtrate, a drop of the CFS was plated on MRS agar and incubated at 37°C under microaerophilic conditions. Sterile paper discs (6 mm diameter, 15 μL absorption) were impregnated with CFS for 3–5 min and dried at 37°C for 4 h. For the preparation of the pathogenic bacteria lawn culture, the revived pathogens were cultured in Mueller‐Hinton broth for 24 h and adjusted to 0.5 McFarland standard turbidity (~8 × 10^8^ CFU/mL). The suspension was then lawn‐inoculated onto Mueller‐Hinton agar plates. Standard antibiotic disks of chloramphenicol, nitrofurantoin, and amoxicillin were used as positive controls. The test was performed in triplicate to assess precision and sensitivity, and the mean diameter of the inhibition zones was recorded after incubation (Shahverdi et al. 2023).

#### 2.4.3 Cell Surface Hydrophobicity

The hydrophobicity on the cell surface of the probiotic strains was determined by microbial adhesion to hydrocarbons, using xylene as the hydrophobic phase. The overnight cultures were collected by centrifuging (6000×*g*, 10 min), and the pellets were washed and resuspended in sterile phosphate‐buffered saline (PBS) to a final concentration of 1 × 10^8^ CFU/mL in 3 mL. The absorbance of the bacterial suspension was recorded at 600 nm (A_0_). A xylene (Merck, Germany) (1 mL) was added, followed by vigorous mixing with a vortex for 2 min. The preparation was incubated at 37°C to allow for phase separation; the aqueous phase was carefully removed, and its absorbance (A_1_) was measured at 600 nm. The percentage of hydrophobicity was calculated using the formula (Mahjoory et al. 2023):

$$\text{Hydrophobicity}\;\left(\%\right)=\left(1-A_{1}/A_{0}\right)\times 100$$

#### 2.4.4 Autoaggregation

The autoaggregation ability of the probiotic strains was determined after a 24‐h incubation (final concentration: 10^7^–10^8^ CFU/mL). Bacterial cells were obtained by centrifugation at 4000×*g* for 10 min, washed twice with sterile PBS, and resuspended in PBS. The resuspensions were incubated for 4 h (without shaking) at 37°C. Absorbance at 600 nm was recorded at times 0 and 4 h (A_0_ and A_*t*_, respectively), and autoaggregation was calculated using the following formula (Soleimani et al. 2023):

$$\text{Autoaggregation}\;\left(\%\right)=\left(A_{0}-A_{t}\right)/A_{t}\times 100.$$

#### 2.4.5 Coaggregation

To study coaggregation, the probiotic strains were co‐cultured with pathogenic *Escherichia coli* ATCC 25922. Cultures were grown at 37°C for 4 h without shaking. Absorbance at 600 nm was measured for each strain separately (A_*p*_ for pathogen, A_*i*_ for isolate), as well as the mixtures (A_mix_). The percent coaggregation was calculated using the following equation (Soleimani et al. 2023):

$$\text{Coaggregation}\;\left(\%\right)=\left[\left(A_{p}+A_{i}\right)/2-A_{\mathrm{mix}}\right]/\left[\left(A_{p}+A_{i}\right)/2\right]\times 100$$

### 2.5 Safety Evaluation

#### 2.5.1 Antibiotic Susceptibility Testing

The infection caused by probiotic strains was assessed based on susceptibility to nine antibiotics that have clinical significance widely used in Iran, including: ciprofloxacin (5 μg), cefixime (5 μg); trimethoprim‐sulfamethoxazole (75 μg), amoxicillin‐clavulanate (10 μg), amoxicillin (25 μg), cephalexin (30 μg), doxycycline (30 μg), and vancomycin (30 μg). Antibiotic resistance was evaluated using the Kovach method with the standard Kirby–Bauer disc diffusion assay. In brief, 1 mL overnight bacterial suspensions for each strain were adjusted to a concentration of 10^7^ CFU/mL and then uniformly plated to MRS agar plates (susceptible). Antibiotic sensitivity discs were placed on the surface of contained inverted caps with semi‐soft agar, and then the plates were incubated at 37°C for 24 h. The opposing diameter of the disc to the inhibition zone was measured with a digital caliper. The individual strain susceptibility or resistance can be interpreted using the specific zone diameter (Nami et al. 2024).

#### 2.5.2 Hemolytic Activity Assay

To evaluate the hemolytic potential of the strains, overnight cultures were streaked on blood agar plates containing 7% (v/v) defibrinated sheep blood. Plates were incubated at 37°C aerobically for 48 h. The hemolytic activity was determined by the absolute or relative presence of clear (β‐hemolysis), greenish (α‐hemolysis), or a lack of (γ‐hemolysis) zones around the colonies (Nami et al. 2024).

### 2.6 Microencapsulation of Potential Probiotic Strains

#### 2.6.1 Preparation of Strains for Microencapsulation

The isolated probiotic strains were grown in MRS broth and incubated at 37°C for 24 h. After incubation, the strains were centrifuged at 6000×*g* for 20 min to pellet the bacterial cells. The supernatant was discarded, and the bacterial pellets were washed three times with sterile phosphate‐buffered saline (PBS, pH 7.0) to remove excess components from the media. The resultant suspension was diluted to 1 × 10^9^ CFU/mL using the same buffer and will be used in the following microencapsulation assessment.

#### 2.6.2 Preparation of Cell–Skim Milk Mixture

All materials and equipment for the process reported in this study were sterilized prior to use. Skim milk powder was dissolved in double distilled water to prepare a 35% (w/w) solution (2 mL of bacterial cell suspension, approximately 2 × 10^9^ CFU/mL, with 28 mL prepared skim milk solution), and allowed to hydrate overnight (0°C–10°C) on a shaker. The probiotic mixture (with skim milk solution) was then stored at 5°C until it was needed.

#### 2.6.3 Microencapsulation Process

A 30 mL volume of the chilled (5°C) skim milk, bacterial cell solution was combined with 400 μL of the rennet stock solution (prepared following the manufacturer's instructions). The combined solution was incubated at 5°C for 60 min to achieve the enzymatic pre‐gelation. Then, 180 μL of a 10% (w/v) sterile calcium chloride solution was added to the combined skim milk–bacterial cell–rennet solution to induce ionic cross‐linking (Nami et al. 2023). Immediately following the addition of calcium chloride, 15 mL of the mixture was transferred into 150 mL of sterile vegetable oil (pre‐cooled to 5°C) contained in a 200 mL Erlenmeyer flask. The emulsion was formed by continuously stirring with a magnetic stir plate set to 500 rpm for 15 min. Continuous stirring reduced the likelihood of gelation during the emulsification process at 5°C. After 15 min of emulsification time, the temperature during emulsification was increased to 40°C. As the emulsion cooled from 40°C to approximately 18°C–20°C, the emulsion droplets transformed into gelled microcapsules. The microcapsules formed in the reaction were separated from the oil phase using slow centrifugation (500×*g*), ensuring gentle conditions. The oil (supernatant phase) was removed, and the pellets were resuspended in double their volumes of double‐distilled water. This wash step was repeated twice more at the same centrifugation speed to ensure that all residual oil traces were eliminated. This method is based on enzymatic gelation and emulsion‐based microencapsulation, where protein‐based microcapsules were obtained and shown to effectively trap viable probiotic cells, as demonstrated by (Heidebach et al. 2009). Rennet‐induced gelation combined with emulsion techniques was chosen for its biocompatibility with milk proteins and high encapsulation efficiency, preserving viability during gastrointestinal transit as validated by Heidebach et al. (2009).

#### 2.6.4 Morphological Observation of Microencapsulated Probiotics

To assess the morphology of the microcapsules, a sample of the microencapsulated probiotic suspension was diluted and then viewed with a light microscope at 1000× magnification. By using a microscope, the general shape and surface characteristics of the capsules could be assessed. The capsule size could not be accurately measured with an optical microscope due to technological limitations. The best way to evaluate the distribution of microcapsule size accurately would likely be X‐ray diffraction (XRD), or it may also be possible with laser diffraction (using a Coulter counter).

#### 2.6.5 Enumeration of Entrapped Bacteria in Microcapsules

To measure the amount of viable entrapped probiotic cells, fresh microcapsules were dispersed in a sterile 1% (w/v) sodium citrate solution (pH≈6) and gently stirred for 10 min at room temperature to dissolve the microcapsules and release encapsulated bacteria. Once completely dissolved, 10‐fold serial dilution steps using sterile phosphate‐buffered saline (PBS) were performed. One milliliter from each dilution was plated on MRS agar using the pour plate method. The plates were incubated for 48 h under anaerobic conditions at 37°C, after which colonies were counted as colony‐forming units (CFU). The total viable counts of cells recovered from capsules were compared to the original cell count used to encapsulate. The encapsulation efficiency (EE) was then calculated, expressed as a percentage of viable cells retained post‐encapsulation relative to the original input.

### 2.7 Animal Model and Experimental Design

#### 2.7.1 Animal Housing Conditions

The study utilized 32 healthy adult Wistar rats (mean weight: 200 ± 30 g) acquired from Tabriz University of Medical Sciences (Wen et al. 2023). Animals were kept in the animal facility of the Biotechnology Department, Agricultural Research and Education Center, under controlled environmental conditions (12 h light/12 h dark cycle, ambient temperature of 20°C–25°C, and relative humidity of 60% ± 5%). The rats had free access to standard laboratory chow and water throughout the study. After a one‐week acclimation period, the animals were randomly assigned to four experimental groups of eight rats using randomization software.

#### 2.7.2 Animal Grouping

The rats were distributed randomly across four experimental groups (*n* = 8 per group), called:

Group 1: A (Normal Control): Healthy rats given a standard diet and 0.2 mL/day of phosphate‐buffered saline (PBS) administered by oral gavage.

Group 2: C (Normal + Probiotics): Healthy rats given a standard diet, which received a selected strain of probiotic at a dose of 1 × 10^9^ CFU/mL/day by oral gavage.

Group 3: B (Diabetic Control): Diabetic rats induced by a single intraperitoneal injection of streptozotocin (STZ), 35 mg/kg body weight. Rats were fed a high‐fat diet (HFD) for 3 weeks prior to STZ injections and continued with a standard diet following STZ injections.

Group 4: D (Diabetic + LAB): Diabetic rats induced by STZ (35 mg/kg BW. i.p.) that received a selected strain of probiotic at a dose of 1 × 10^9^ CFU/mL/day by oral gavage. The rats were fed a high‐fat diet for 3 weeks prior to the induction of diabetes and were placed on a standard diet throughout the study intervention period.

#### 2.7.3 Preparation of High‐Fat Diet (HFD)

Rats belonging to designated groups received a high‐fat diet (HFD) during the three‐week pre‐diabetes induction period, with a daily average consumption of 30 g per rat. The HFD was made by combining 1 kg of standard rodent chow with 200 g of rendered animal fat (tallow) and 200 g of clarified animal fat (ghee). The standard diet contained the following nutritional components: crude protein (24%), crude fat (4.5%–5.5%), crude fiber (3.5%–4%), ash (max. 10%), calcium (0.95%), phosphorus (0.65%–0.70%), moisture (10%), salt (0.5%–0.55%), lysine (1.25%), methionine (0.33%), methionine + cysteine (0.63%), threonine (0.72%), tryptophan (0.25%), metabolizable energy (16.16–17 MJ/kg). We added a small amount of distilled water to achieve homogenization and properly mixed the sample. We then extruded the paste through a funnel to form small pellets, followed by drying the pellets in an oven. After preparation, we stored the HFD pellets in a hygienic environment and fed the rats daily at approximately noon in 30‐g measured quantities.

#### 2.7.4 Induction of Diabetes in Rats

Rats were induced to become diabetic after previous work using streptozotocin (STZ) as above. The metabolic changes caused by STZ were similar to changes occurring in human type 2 diabetes mellitus. Prior to the use of STZ, the animals were fed a high‐fat diet for 3 weeks, that is, each animal consumed 1 kg of regular chow modified from adding to each 1 kg of regular chow 200 g of clarified animal butter (ghee) and 200 g of sheep tallow, as well as giving each animal the chow mixture of the 1 kg, each animal was given the chow mixture homogenized now in the form of pellets in a fashion they would get the same daily. STZ (Sigma‐Aldrich, USA) was freshly dissolved in sterile distilled injectable water. After the three‐week high‐fat diet, the animals were injected using insulin syringes with a single intraperitoneal injected dose of 35 mg/kg body weight. The rats were fasted for 24 h, after which diabetes was confirmed by measuring their fasting blood glucose levels. This was achieved by making a small incision on the tail using a sterile lancet and obtaining a drop of blood for analysis using a glucometer (Easy Gluco, Infopia, Korea). Animals were designated as irremediably diabetic by fasting blood glucose levels > 250 mg/dL. These animals were included in the experimental diabetic groups. The low‐dose STZ (35 mg/kg) following the 3‐week HFD regimen mimics human T2DM pathogenesis by inducing partial β‐cell dysfunction and insulin resistance, rather than complete β‐cell ablation seen in high‐dose models.

#### 2.7.5 Daily Oral Gavage

The probiotic bacteria protected within microcapsules were fresh on the day of administration and stored and prepared daily at a temperature of 4°C. Each rat received a dose of 1 × 10^9^ CFU/mL of the probiotic suspension using a 2 mL PRS sterile syringe and gavage needle. Modified gavage in each rat was conducted individually and at the same time each day for the duration of the intervention.

#### 2.7.6 Streptozotocin (STZ) Injection

Streptozotocin (STZ) is a common and widely used compound to induce both type 1 and type 2 experimental diabetes using animal models. STZ can be given intravenously (IV), intraperitoneally (Khongtan et al. 2023), and subcutaneously. The IV dosing range, which is typically used to induce type 1 diabetes, is between 40 and 65 mg/kg body weight. Higher doses of STZ are needed to induce type 1 diabetes via the IP route successfully. The effects on blood glucose and insulin levels are induced by the possibility that STZ causes significant functional damage to pancreatic beta cells. Streptozotocin interrupts glucose oxidation as well as the synthesis and secretion of insulin. Streptozotocin gets preferentially taken up by pancreatic beta cells using the glucose transporter GLUT2, allowing STZ to enter these cells and display cytotoxic effects. The selective toxicity of STZ in beta cells has been linked to DNA damage, primarily through alkylation. Furthermore, down‐regulation of the GLUT2 expression may alleviate STZ‐induced cytotoxicity. The destruction and death of beta cells can largely be attributed to STZ‐induced DNA strand breaks and damage, which also leads to irreversible hyperglycemia and diabetes (Akbarzadeh et al. 2007).

#### 2.7.7 Blood Sampling From Rats

At the end of the 8‐week treatment phase (including a pre‐treatment 3‐week acclimation phase), the animals were ready for blood sampling. All items (and equipment) used in the blood sampling process were sterilized to maintain sterility and laboratory standards. Due to difficulties in accessing and low volumes possible from the needed route to obtain tail vein withdrawal blood, challenges were encountered. Because of this, rats were anesthetized at the suggested dose of anesthetic (isoflurane), and blood samples were taken via retro‐orbital sinus puncture and cardiac puncture under aseptic conditions to obtain sufficient volumes for serum analysis. Blood samples were centrifuged at 3000×*g* for 5 min to obtain serum. The serum was stored and analyzed for biochemical and immunological assays. All handling of animals was conducted by institutional ethical guidelines and approved protocols for animal care.

#### 2.7.8 Measurement of Food and Water Intake

In order to measure daily food and water consumption, a set amount of 30 g of feed was provided to each rat each day at the same time daily. After 24 h, the remaining food was weighed using an exact digital scale, and the amount eaten was calculated and recorded in grams. Water intake was measured simultaneously by using calibrated drinking bottles in which the volume consumed each day was also recorded in milliliters for each rat. These measures were then completed daily across the experimental period to assess changes in food intake under different experimental conditions.

#### 2.7.9 Fasting Blood Glucose Measurement

For accurate fasting blood glucose (FBG) evaluation, all rats were fasted overnight for at least 8 h. The food was removed from the cages the previous night, and then FBG measures were taken early the next morning. Using a sterile lancet, a small incision was made in the tail vein to collect a drop of blood, which was then placed on a test strip connected to a calibrated glucometer. Values of the blood glucose were captured in mg/dL. FBG was measured in four instances during the study timeframe, prior to and following diabetes induction, to assess glycemic changes throughout the intervention.

#### 2.7.10 Serum Insulin Measurement

Following blood collection and serum separation, the samples were immediately transported to the biochemistry laboratory for the determination of serum insulin concentrations.

#### 2.7.11 Measurement of Interleukins and TNF‐α

Serum levels of IL‐1, IL‐6, and TNF‐α were determined using enzyme‐linked immunosorbent assay kits (Zheng et al. 2020). In brief, standards, serum samples, and biotinylated anti‐cytokine antibodies were added to microplate wells that had been previously coated with capture antibodies. After incubation, any unbound biotinylated antibodies were washed away, and streptavidin‐HRP was added to bind the biotinylated antibodies. Following a second wash step, the TMB substrate solution was added to the wells, and a colorimetric reaction occurred. The color catalyzed from blue to yellow depending on cytokine concentration. Absorbance was ultimately read using a microplate reader, and concentrations were calculated using standard curves. A certified biochemistry private laboratory completed all analyses.

### 2.8 Statistical Analysis

All experimental data are expressed as mean ± standard deviation (Todorov and Dicks 2005). Statistical analysis was performed using SPSS version 26.0 (IBM Corp., Armonk, NY, USA). The normality of data distribution was confirmed using the Shapiro–Wilk test and the homogeneity of variances was checked using Levene's test before conducting parametric analysis. Comparisons of means among multiple groups were analyzed preliminarily using one‐way analysis of variance (ANOVA) followed by Tukey's honestly significant difference (HSD) post hoc test for pairwise comparison of differences. If data did not meet parametric assumptions, comparisons were made using the Kruskal–Wallis test, followed by Dunn's post hoc test with correction. Graphs were made using GraphPad Prism version 9 (GraphPad Software Inc., USA). Parametric (ANOVA/Tukey) or non‐parametric (Kruskal‐Wallis/Dunn) tests were applied based on normality and variance checks to ensure rigorous and appropriate statistical inference.

## 3 Results and Discussion

### 3.1 Biochemical and Molecular Identification

From 15 traditional cheese samples, 40 bacterial isolates were initially obtained, of which 15 were identified as presumptive LAB based on biochemical screening (detailed in Section 2.2). The detailed biochemical profiles of these 15 isolates, including Gram staining, catalase, oxidase, citrate utilization, indole, motility, H_2_S production, nitrate reduction, and carbohydrate fermentation (20 substrates), are summarized in Table S1. All isolates were Gram‐positive, catalase‐negative, oxidase‐negative, non‐motile, and did not produce H_2_S or indole, confirming their classification as LAB. Strains D1 and D2 exhibited fermentation patterns identical to reference *Lactiplantibacillus* species, supporting their selection for molecular identification and further probiotic evaluation.

These 15 isolates were subjected to in vitro probiotic screening assays, including acid and bile salt tolerance, hydrophobicity, autoaggregation, and antimicrobial activity. Among them, two strains, D1 and D2, consistently exhibited the most promising probiotic characteristics across all parameters. Consequently, they were selected for molecular identification.

Molecular identification via 16S rRNA gene sequencing confirmed that strain D1 belonged to *Lactiplantibacillus pentosus* and strain D2 to *Lactiplantibacillus plantarum*. Owing to their superior probiotic traits, both strains were selected for microencapsulation and subsequent in vivo evaluation in streptozotocin‐induced diabetic rats.

### 3.2 In Vitro Evaluation of Probiotic Properties

All 15 biochemically confirmed LAB isolates were subjected to in vitro probiotic screening based on internationally accepted guidelines FAO/WHO (Joint 2002), including tests for acid and bile salt tolerance, hydrophobicity, autoaggregation, coaggregation with *E. coli* , and antimicrobial activity against common gastrointestinal pathogens (Table 1).

**TABLE 1:** Probiotic characterization of isolated strains based on stress tolerance, cell surface properties, and antimicrobial activity.

Isolated strains	Tolerance after 4 h at 0.3% bile	Tolerance after 3 h at pH 2.5	Hydrophobicity (%)	Autoaggregation (%)	Coaggregation with *E. coli* (%)	Antimicrobial activity against			
						*S. aureus*	*E. coli*	*L. monocytogenes*	*S. typhimurium*
D1	64.63 ± 1.22^a^	71.14 ± 1.17^a^	63.15 ± 0.89^b^	68.28 ± 1.40^a^	52.02 ± 0.63^a^	S (22 mm)	M (17 mm)	S (27 mm)	S (23 mm)
D2	65.14 ± 0.94^a^	70.25 ± 1.00^a^	65.35 ± 1.08^a^	66.32 ± 1.48^a^	51.15 ± 0.89^ab^	S (21 mm)	M (19 mm)	M (20 mm)	S (23 mm)
D3	32.81 ± 1.29^de^	34.27 ± 0.78^e^	23.16 ± 0.65^g^	21.15 ± 0.73^g^	19.08 ± 0.59^f^	M (14 mm)	S (21 mm)	M (13 mm)	M (17 mm)
D4	42.91 ± 0.88^b^	45.39 ± 0.45^b^	20.45 ± 0.47^h^	19.53 ± 0.54^gh^	17.43 ± 0.58^g^	— (0 mm)	M (11 mm)	W (7 mm)	W (9 mm)
D5	32.07 ± 0.62^e^	37.31 ± 0.47^cd^	13.23 ± 0.57^k^	8.56 ± 0.37^l^	5.41 ± 0.29^j^	W (8 mm)	M (19 mm)	— (0 mm)	M (13 mm)
D6	13.15 ± 0.47^i^	14.61 ± 0.36^h^	7.72 ± 0.24^l^	6.33 ± 0.32^m^	6.31 ± 0.24^j^	M (14 mm)	— (0 mm)	W (9 mm)	W (7 mm)
D7	7.18 ± 0.38^j^	8.59 ± 0.34^i^	15.69 ± 0.27^j^	13.11 ± 0.36^k^	11.29 ± 0.25^i^	— (0 mm)	M (13 mm)	W (7 mm)	W (6 mm)
D8	27.38 ± 0.46^f^	28.49 ± 0.40^f^	17.38 ± 0.29^i^	18.59 ± 0.42^hi^	22.87 ± 0.44^e^	M (11 mm)	— (0 mm)	— (0 mm)	M (13 mm)
D9	34.37 ± 0.50^cd^	29.46 ± 0.42^f^	33.29 ± 0.56^f^	34.25 ± 0.47^e^	35.30 ± 0.59^d^	W (4 mm)	M (11 mm)	W (9 mm)	— (0 mm)
D10	42.13 ± 0.65^b^	38.52 ± 0.46^c^	44.16 ± 0.55^e^	40.37 ± 0.73^d^	41.26 ± 0.40^c^	— (0 mm)	M (17 mm)	W (7 mm)	W (5 mm)
D11	16.32 ± 0.56^h^	18.44 ± 0.11^g^	17.53 ± 0.39^i^	15.55 ± 0.36^j^	13.98 ± 0.41^h^	M (13 mm)	— (0 mm)	— (0 mm)	M (11 mm)
D12	22.16 ± 0.47^g^	18.84 ± 0.35^g^	22.99 ± 0.47^g^	25.05 ± 0.57^f^	23.86 ± 0.38^e^	W (9 mm)	M (12 mm)	W (9 mm)	W (7 mm)
D13	42.21 ± 0.54^b^	38.90 ± 0.36^c^	46.22 ± 0.35^d^	43.86 ± 0.40^c^	41.05 ± 0.41^c^	M (12 mm)	M (17 mm)	— (0 mm)	W (9 mm)
D14	35.18 ± 0.59^c^	36.43 ± 0.42^d^	50.05 ± 0.43^c^	47.57 ± 0.37^b^	50.35 ± 0.42^b^	— (0 mm)	M (19 mm)	W (7 mm)	M (14 mm)
D15	22.17 ± 0.69^g^	13.05 ± 0.15^h^	14.68 ± 0.25^jk^	17.08 ± 0.48^ij^	18.60 ± 0.38^fg^	M (14 mm)	— (0 mm)	W (9 mm)	W (7 mm)

*Note:* Antibacterial activity: S = strong (≥ 20 mm), M = moderate (10–19 mm), W = weak (≤ 10 mm), — = no inhibition zone. Values are means ± SD of triplicates. Different superscript letters (a–c) in the same column indicate significant differences (*p* < 0.05).

D1 and D2 showed the highest survival under simulated gastrointestinal conditions (pH 2.5 and 0.3% bile). D1 showed 64.63% ± 1.22% survival in bile and 71.14% ± 1.17% at pH 2.5, while D2 had comparable rates of 65.14% ± 0.94% and 70.25% ± 1.00%. Conversely, isolates D6 and D7 exhibited poor viability, indicating weak probiotic resilience. D1 and D2 exhibited the highest cell surface hydrophobicity (63.15% and 65.35%) and autoaggregation (68.28% and 66.32%), indicating strong adhesion potential to intestinal epithelium and capability for colonization. In contrast, D5 and D6 had low values in both assays (< 10%), indicating limited colonization potential. Coaggregation values for D1 and D2 were 52.02% and 51.15%, respectively, supporting their ability to compete with pathogens and contribute to gut barrier defense. D6 and D5 had negligible coaggregation. Both strains showed strong inhibition against *S. aureus, E. coli, L. monocytogenes*, and *S. typhimurium* . D1 demonstrated inhibition zones of ≥ 22 mm for all tested pathogens; D2 also showed notable inhibition, especially against *E. coli* and *S. typhimurium* . Weak or no activity was observed in isolates D6, D7, and D11.

Taken together, isolates D1 and D2 consistently demonstrated superior probiotic characteristics across all evaluated parameters and were thus selected as the most promising strains for subsequent molecular identification, microencapsulation, and in vivo investigation. These results strongly support the probiotic potential of strains D1 and D2 based on internationally established in vitro screening criteria (Joint 2002). Their high survival rates under acidic (pH 2.5) and bile salt (0.3%) conditions indicate their likely resilience during gastrointestinal transit, which is a fundamental trait for probiotic functionality. Moreover, their elevated cell surface hydrophobicity and autoaggregation abilities suggest enhanced adhesion capacity to intestinal epithelial cells, facilitating colonization and pathogen exclusion (Collado et al. 2008).

Both strains also exhibited strong coaggregation with *Escherichia coli* , reinforcing their potential to form protective biofilms and interfere with pathogen adherence. Antimicrobial assays demonstrated that D1 and D2 possess broad‐spectrum inhibitory effects against *S. aureus* , *E. coli, L. monocytogenes*, and *S. typhimurium* , which may be attributed to the secretion of organic acids, hydrogen peroxide, or bacteriocin‐like compounds, as described for other *Lactobacillus* species (Todorov and Dicks 2005). These strain‐dependent differences in inhibitory spectrum highlight the unique metabolic capabilities of D1 and D2.

In summary, D1 and D2 exhibited the most promising probiotic characteristics and were selected for microencapsulation and subsequent in vivo analysis—including immunological safety tests—to evaluate their antidiabetic and anti‐inflammatory efficacy in a rat model.

### 3.3 Safety Assessment of Isolated Probiotic Strains

Table 2 indicates the observed profile of hemolytic ability and resistance among the isolated strains. Both selected strains (D1, and D2) exhibited a γ‐hemolytic profile, which indicated no hemolytic activity and a non‐pathogenic nature that is appropriate for probiotic use. The resistance profiles of each strain differed. Strain D1 was resistant to cefixime and vancomycin (0.00 and 11.24 mm, respectively) but also had very high inhibition of amoxicillin‐clavulanic acid (24.47 mm) and had some inhibition against ciprofloxacin (19.23 mm) (Table 2). D2 was highly susceptible to amoxicillin (26.32 mm), doxycycline (37.16 mm), AMC (31.06 mm), and showed moderate resistance against vancomycin (8.13 mm).

**TABLE 2:** Safety characteristics of the isolated probiotic strains based on antibiotic susceptibility and hemolytic activity.

Strains	Antibiotics								Hemolysis
	CFM (mm)	AMX (mm)	D (mm)	SXT (mm)	CP (mm)	CN (mm)	AMC (mm)	V (mm)	
D1	0.00 ± 0.00	18.36 ± 1.69	10.46 ± 1.18	14.52 ± 1.46	19.23 ± 2.01	11.25 ± 1.09	24.47 ± 1.84	11.24 ± 1.14	γ (non‐hemolytic)
D2	13.17 ± 1.53	26.32 ± 1.43	37.16 ± 1.43	10.31 ± 1.27	23.41 ± 2.02	14.54 ± 1.42	31.06 ± 2.04	8.13 ± 1.20	γ (non‐hemolytic)

*Note:* Interpretation according to CLSI (2013) standards.

Abbreviations: AMC, amoxicillin‐clavulanic acid; AMX, amoxicillin; CFM, cefixime; CN, cephalexin; CP, ciprofloxacin; D, doxycycline; SXT, trimethoprim‐sulfamethoxazole; V, vancomycin.

The lack of hemolytic activity in all isolates represents a significant safety factor, ensuring their potential use in food or therapeutic applications (Salek et al. 2023; Soleimani et al. 2023). The γ‐hemolytic activity indicates that the strains do not lyse red blood cells, which suggests less concern for cytotoxicity or pathogenicity. Variable antibiotic susceptibility profiles are typical for species of *Lactobacillus* and *Pediococcus*, which present intrinsic and acquired resistance (Gueimonde et al. 2013). The resistance to vancomycin exhibited by strain D1 was corroborated by previous work, which showed that many lactic acid bacteria possess intrinsic resistance to vancomycin because they lack the D‐Ala‐D‐Lac (Mathur and Singh 2005). Importantly, none of the strains had multi‐drug resistance patterns of concern from a health standpoint, as stipulated in the EFSA (European Food Safety Authority) guidelines. Thus, the combination of neither hemolytic behavior nor patterns of antibiotic susceptibility provides reasonable justification to consider D1, and D2 as safe probiotic candidates for further research applications in diabetic and anti‐inflammatory models with animals.

### 3.4 Microencapsulation Performance

Evaluating the encapsulation efficiency, by looking at the viable cell counts before and after the encapsulation across two independent trials. The results for the first trial indicated that the initial cell density of about 9.02 log CFU/mL exhibited a slight increase to 9.2 log CFU/mL following encapsulation, demonstrating no meaningful loss of viable cells and possibly improved dispersion and/or recovery during processing. In the second trial, a minimal decrease was observed, from a log density of 10–9.8 log CFU/mL, indicating again that there was no significant loss of viable cells when encapsulating the microcapsules.

Examining the capsules microscopically indicated that the resultant microcapsules produced by both enzymatic gelation and emulsification were spherical, thus supporting the visual consistency and uniformity of these methods. The shape of these microcapsules is considered advantageous for stability, protection against environmental stressors, and the expected release of probiotic bacteria in gastrointestinal or digestive conditions.

Overall, these results demonstrate that the microencapsulation technique employed in this study was effective in preserving bacterial viability across various trials, with minimal loss in viable cell counts. The maintenance of viable cell counts in both trials was consistently high, indicating that this method may be suitable for probiotic delivery, ensuring consistent probiotic levels. For instance, maintaining viable cell counts is crucial for ensuring the functional efficacy of probiotic bacterial products in therapeutic settings, such as those used in diabetes management.

### 3.5 Fasting Blood Glucose (FBG)

Table 3 summarizes the results. One‐way ANOVA revealed significant differences in post‐intervention FBG (*F*(3,28) = 498.23, *p* < 0.001). Tukey's post hoc test confirmed Group D had significantly lower FBG than Group B (*p* < 0.001), with no significant difference between Groups A and C (*p* = 0.78). Baseline FBG levels were similar across all four groups (normal control (A), diabetic control (B), non‐diabetic probiotic‐treated (C), and diabetic probiotic‐treated (D)), with no significant differences detected (*p* < 0.05). Therefore, homogeneity across groups was established before the intervention. After the STZ injection and induction of diabetes, there was a significant spike in the FBG levels in the diabetic groups (B and D) at 48 h post‐injection, indicating successful induction of hyperglycemia. The average FBG for group B increased from approximately 75.12 –317.14 mg/dL, and the average in group D rose to 200.75 mg/dL. The vast difference between groups B and D was statistically significant (*p* < 0.05) based on Fisher's test, concluding that probiotic treatment significantly reduced the overall increase in blood glucose levels for diabetic rats.

**TABLE 3:** Fasting blood glucose (FBG) levels (mg/dL) in four experimental groups before and after STZ‐induced diabetes.

Group	Before STZ	After STZ
Group A (Normal Control)	75.84 ± 1.25^a^	76.62 ± 1.27^a^
Group B (Diabetic Control)	75.12 ± 2.01^a^	317.14 ± 2.95^c^
Group C (Normal + Probiotic)	74.35 ± 1.36^a^	74.75 ± 1.49^a^
Group D (Diabetic + Probiotic)	75.54 ± 1.97^a^	200.75 ± 2.41^b^

*Note:* Values are mean ± SD (*n* = 8). Group A, Healthy rats + PBS; Group B, STZ‐induced diabetic + PBS; Group C, Healthy +1 × 10^9^ CFU/day probiotics; Group D, STZ‐induced diabetic +1 × 10^9^ CFU/day probiotics. Different superscript letters in the “After STZ” column indicate significant differences (Tukey's test, *p* < 0.05).

The rats in Groups A and C displayed relatively stable FBG means (76.62 and 74.75 mg/dL, respectively) with no significant change and with no treatment effect in non‐diabetic (already healthy) rats. The indication that the oral administration of these selected probiotic strains attenuated hyperglycemia for STZ‐induced diabetic rats, which was not determined to be the case in normoglycemic rodents, further suggests an obvious therapeutic advantage of potential probiotics used only in response to diabetic pathology. This reinforces the notion that probiotics can impact glycemic levels in STZ‐induced diabetic rats and is consistent with the hypothesis that probiotics may influence glycemic levels through the modulation of gut microbiota or improvement of metabolic pathways in non‐diabetic rodents.

The relative reduction in hyperglycemia of the probiotic‐treated diabetic rats (group D) compared to the untreated diabetic controls (group B) is clear evidence that probiotics administered in the complete diabetes model post‐STZ were protective against hyperglycemia. After STZ injection, both groups exhibited increased fasting blood glucose (FBG) levels. However, the substantially lower FBG levels observed in group D (200.75 vs. 317.14 mg/dL in group B) imply that the probiotic reduced hyperglycemia in group D to the extent that it did not return to normoglycemia but limited the severity of hyperglycemia with STZ injection. This observation is consistent with previous studies that have shown that specific probiotic strains (primarily *Lactobacillus* and *Bifidobacterium*) can restore hyperglycemia through modulation of gut microbiota and systemic inflammatory pathways (Bagarolli et al. 2017; Yadav et al. 2013). The reduction of glycemic burden could also be attributed to improved intestinal barrier function, reduced endotoxin‐mediated inflammatory effects, and increased production of SCFAs, which enhances insulin sensitivity (Canfora et al. 2019; Hemarajata and Versalovic 2013).

Although the present study did not distinguish between encapsulated and non‐encapsulated probiotics, the beneficial effect demonstrated would suggest that even non‐encapsulated forms of delivery may provide metabolic benefits in a diabetic context. These results may be contingent on the presence of metabolic dysfunction since no meaningful change was found in groups A and C (normoglycemic rats), which supports the probiotics' selective efficacy in disease‐altering situations. Collectively, these results maintain the support of probiotics acting as supplementary therapeutic agents in diabetes mellitus (type 2) treatment through a dampening effect on the progression of hyperglycemia but not restoring any established glycemic abnormalities. Research exploring dose–response relationships, type of encapsulation, and microbiome shifts is needed for future research into clinical applications.

While mechanistic pathways were not directly explored in the current study, the reduction of hyperglycemia noted in the probiotic‐treated rats may be associated with mechanisms known to be associated with probiotics, including gut microbiota modulation, enhancement of intestinal barrier integrity, and improvement of systemic inflammation described in the literature (Canfora et al. 2019; Hemarajata and Versalovic 2013). Although these pathways were not measured in this study, they provide a plausible explanation for the observed improvement in glycemic control.

### 3.6 Body Weight Changes

The ending body weights for the rats in the experimental groups can be found in Table 4. ANOVA indicated significant group effects on final body weight (*F*(3,28) = 18.91, *p* < 0.001). Post hoc analysis showed Group D retained significantly higher weight than Group B (*p* = 0.002), with no significant difference between A and C (*p* = 0.12). The starting weight of the rats at the beginning of the study was approximately 200 ± 30 g, and there were no statistical differences between groups. By the end of the 8‐week study, the rats weighed significantly different amounts. The normal control group (A) had an average body weight of 240 g, indicating that under normal conditions, the rats achieved their normal and healthy weight. The non‐diabetic group with probiotic treatment (group C) achieved a slight increase, with a final average body weight of 250 g, possibly displaying a modest growth‐promoting effect of probiotics on normoglycemic animals. Diabetic control rats (B) significantly decreased in total body weight, with a final average of 215 g, which is to be expected, given the catabolic actions seen in diabetic patients. Diabetic rats that were given probiotic treatment (group D) were able to hold onto their weight significantly better, achieving an average final weight of 245 g. Furthermore, statistical analysis revealed a significant difference in body weight between rats in groups B and D (*p* < 0.05), suggesting that probiotic treatment in diabetic rats may mitigate weight loss associated with the diabetes condition.

**TABLE 4:** Body weight of rats (g) in each experimental group at the start and end of the 8‐week study.

Group	Initial (week 0)	Final (Week 8)
Group A (Normal Control)	200 ± 1.96^a^	240.88 ± 2.03^a^
Group B (Diabetic Control)	200 ± 2.24^a^	214.38 ± 2.67^b^
Group C (Normal + Probiotic)	200 ± 2.04^a^	250 ± 2.00^a^
Group D (Diabetic + Probiotic)	200 ± 1.78^a^	245.5 ± 1.6^a^

*Note:* Values are mean ± SD (*n* = 8). Group definitions as in Table 3. Different superscript letters in the “Final” column indicate significant differences (Tukey's test, *p* < 0.05).

The association between weight loss and uncontrolled diabetes mellitus is a well‐established problem, marked by significant changes in specific endpoints. For example, in diabetes mellitus, there is a shift from glucose‐directed energy use to lipid‐ and protein‐based energy use due to insulin deficiency, rendering it impossible to regulate energy effectively. In this study, all of the STZ‐induced diabetic rats had a significant decrease in body weight (Table 4) at the end of the study (Week 4); on the other hand, the diabetic rats in the immune probiotic group (group D) showed body weight at close to if not the same as the normal rats. This reinforces previous reports on the impact of probiotics on body weight stabilization and elucidates the mechanisms involved. Lactic acid bacteria, such as *Lactobacillus* (3), are beneficial to the gut microbiota, with a variety of functions, including shifting gut microbiota composition, promoting the production of SCFAs, and decreasing intestinal inflammation and absorption (Li et al. 2020). SCFAs, such as butyrate, have been reported to regulate energy homeostasis and catabolic stress in diabetes‐induced metabolic models (Cani et al. 2007; Everard et al. 2013). The considerable body weight impact of treatment group D, as well as the previously noted effect of saffron, may offer additional support for dietary supplementation with probiotics as an adjunctive strategy for type 2 diabetes.

### 3.7 Serum Insulin Levels

**FIGURE 2:**
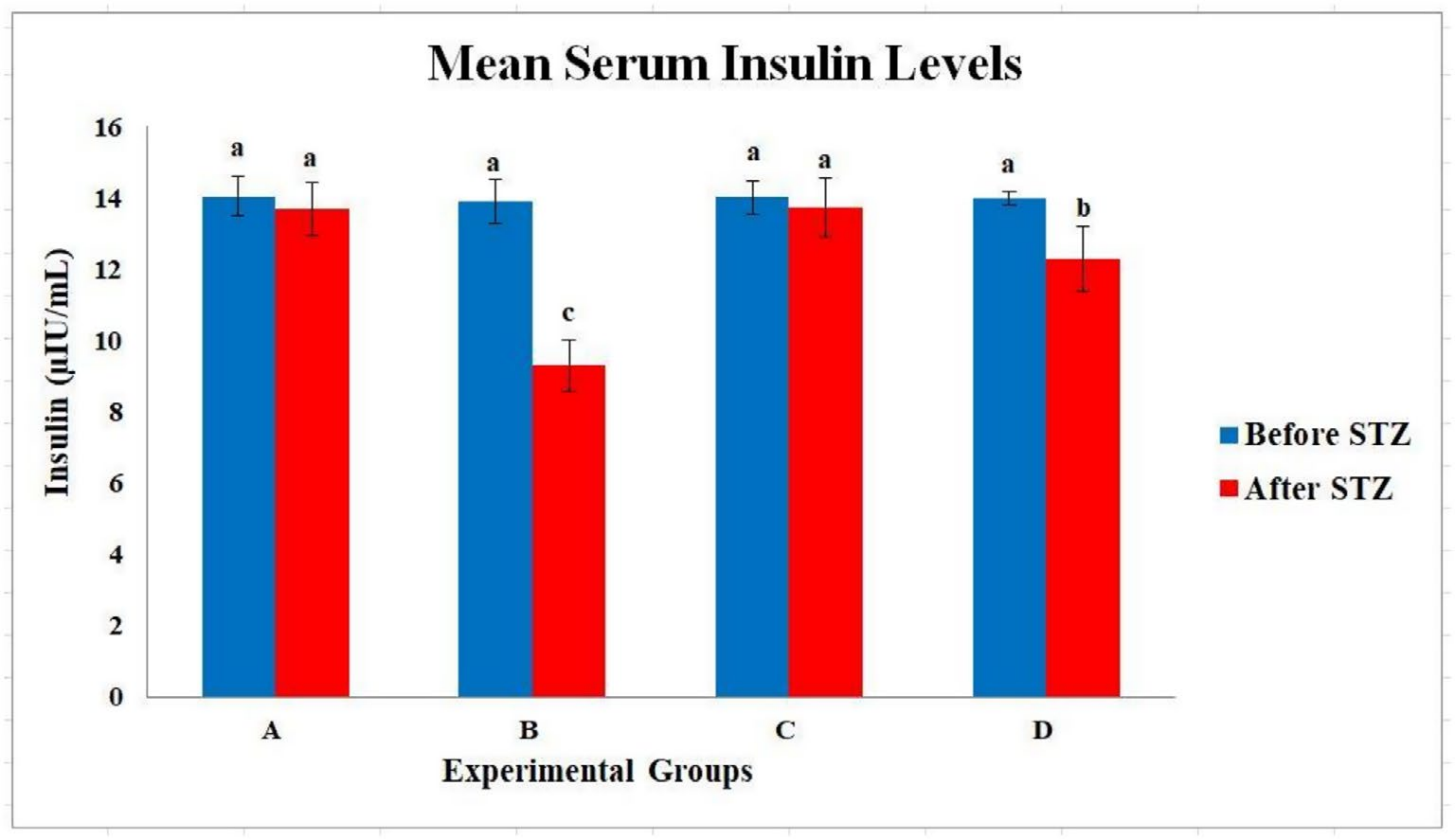
Mean serum insulin levels before and after treatment in each group. This bar chart compares insulin levels (μIU/mL) measured before and after the intervention across all four experimental groups. It demonstrates a significant decrease in serum insulin in the diabetic control group and a moderate recovery in the diabetic group supplemented with probiotics. (A) Normal control, (B) Diabetic control, (C) Normal + probiotic, and (D) Diabetic + probiotic. Error bars represent standard deviation.

The summary of fasting serum insulin levels is illustrated in Figure 2, and the data are summarized in Table 5. Pre‐ and post‐intervention fasting serum insulin levels were measured in all four groups. Before sham surgery and diabetes induction, mean fasting serum insulin levels were statistically similar among groups (A: 14.06 μIU/mL; B: 13.90 μIU/mL; C: 14.03 μIU/mL; D: 14.00 μIU/mL) with no significant differences (*p* < 0.05) confirming the groups were homogenous prior to treatment. One week post‐administration of STZ and 7‐week administration of probiotics, the mean serum insulin level in the diabetic control group (B) was substantially decreased (9.3 ± 0.9 μIU/mL), which reflects the loss of serum insulin due to STZ‐induced cell death in the pancreas. The mean serum insulin level in the diabetic probiotic group (D) was statistically higher (12.3 ± 1.0 μIU/mL), demonstrating a protective effect of the probiotic administration (*p* < 0.05). Insulin levels in the normal probiotic intervention group (C) remained statistically similar to the healthy control (A) (15.1 vs. 15.0 μIU/mL, respectively), with no significant differences between groups (*p* < 0.05). A one‐way ANOVA and Tukey's post hoc tests confirmed a statistically significant difference (*p* < 0.01) in the post‐treatment insulin levels between B and D. At the same time, there were no significant differences between groups A and C.

**TABLE 5:** Mean serum insulin concentrations (μIU/mL) and standard deviations in the four experimental groups before and after intervention.

Group	Before STZ	After intervention
Group A (Normal Control)	14.06 ± 0.28^a^	13.69 ± 0.20^a^
Group B (Diabetic Control)	13.91 ± 0.20^a^	9.30 ± 0.90^c^
Group C (Normal + Probiotic)	14.03 ± 0.21^a^	13.74 ± 0.18^a^
Group D (Diabetic + Probiotic)	14.00 ± 0.21^a^	12.30 ± 1.00^b^

*Note:* Values are mean ± SD (*n* = 8). Group definitions as in Table 3. Different superscript letters in the “After Intervention” column indicate significant differences (Tukey's test, *p* < 0.05).

The changes observed in serum insulin levels provide strong evidence in favor of the theoretical effect of probiotic treatment on reversing β‐cell dysfunction induced by T2DM. As was expected, STZ‐induced diabetic rats (group B) had a significantly lower serum insulin level, which is consistent with earlier research on STZ showing that DNA alkylation, oxidative stress, and apoptosis induced by STZ have damaging effects on pancreatic β‐cells that lead to reduced insulin secretion (Szkudelski 2001). However, diabetic rats that received probiotic treatment (group D) had a serum insulin level that was significantly greater than those in group B and indicated that there were still some functional β‐cells producing insulin in this group. The evidence for the probiotic effect on β‐cell function can be attributed to the anti‐inflammatory and immunomodulatory mechanisms of the Lactobacillus species used in this study. Probiotics are well known to improve gut barrier function by decreasing lipopolysaccharide (LPS) translocation, decreasing proinflammatory cytokines (like TNF‐α and IL‐6) associated with insulin signaling resistance, and preventing apoptosis of β‐cells (Canfora et al. 2019; Li et al. 2020).

Furthermore, SCFAs formed from probiotics metabolism, especially butyrate, may stimulate glucagon‐like peptide‐1 (GLP‐1) secretion. GLP‐1 has a role in glucose homeostasis and insulinotropic properties (Zheng et al. 2024). The use of encapsulated probiotics was likely advantageous for maintaining bacterial viability and enhancing colonization efficiency, allowing for more stable modulation of the gut microbiota and associated metabolic benefits (Burgain et al. 2011). Notably, there was no remarkable difference in insulin levels between the standard control (A) and non‐diabetic probiotic‐treated (C) groups, which confirmed that probiotic administration was safe in non‐diabetic conditions with normal glycemic levels. This is evidence for the specific efficacy of probiotics administered to pathological conditions, such as T2DM when compared to nonspecific endocrine stimulation. Collectively, our data suggest that the oral administration of encapsulated Lactobacillus strains may help prevent insulin levels from rising in diabetic participants, which is a potential complementary strategy for T2DM.

### 3.8 Serum Cytokine Profiles

According to Table 6, inflammatory cytokines demonstrated differences between experimental groups. ANOVA detected significant differences for IL‐1β (*F*(3,28) = 156.31, *p* < 0.001), IL‐6 (*F*(3,28) = 89.42, *p* < 0.001), and TNF‐α (*F*(3,28) = 112.74, *p* < 0.001). Tukey's test confirmed reductions in Group D versus B for all cytokines (*p* < 0.001) and no differences between A and C (*p* > 0.05). For example, IL‐1 was considerably higher in the diabetic control group (D), at 92.3 ± 10.6 pg/mL, than in the normal control (A), at 13.8 ± 1.2 pg/mL. Probiotic treatment reduced IL‐1 in the diabetic rodents (B) group to a statistically significant level of 35.0 ± 6.1 pg/mL (*p* < 0.01). The normal group receiving probiotics (C) did not differ statistically from group A. IL‐6 concentrations were also highest for the diabetic control group (35.1 ± 4.7 pg/mL), while a significant reduction was observed in the diabetic + probiotics (D) group, 29.8 ± 1.4 pg/mL; *p* < 0.01. There was no statistically significant difference between the A and C groups (17.4 ± 1.4 vs. 14.2 ± 1.2 pg/mL, respectively).

**TABLE 6:** Mean and standard deviation of IL‐1, IL‐6, and TNF‐α in experimental groups.

Group	IL‐1	IL‐6	TNF‐I
Group A (Normal Control)	13.8 ± 1.2	17.4 ± 1.4	178 ± 16.4
Group B (Diabetic Control)	92.3 ± 10.6	35.1 ± 4.7	392 ± 22.4
Group C (Normal + Probiotic)	13.2 ± 1.2	14.2 ± 1.2	156.5 ± 10.8
Group D (Diabetic + Probiotic)	35 ± 6.1	29.8 ± 1.4	288 ± 21.5

*Note:* Values are mean ± SD (*n* = 8). Group definitions as in Table 3.

The increases in IL‐1, IL‐6, and TNF‐α in the diabetic rats were in line with the inflammatory characteristics of causing type 2 diabetes. The proinflammatory cytokines released by chronic hyperglycemia and continued insulin resistance, which curtail glucose uptake into beta‐cells, will all lead to the activation and production of proinflammatory cytokines. The design of streptozotocin‐induced diabetes presented herein has been previously noted, with streptozotocin leading to an increase in circulating inflammatory mediators initiated by oxidative stress, as well as dysregulation of immune pathways, resulting in metabolic dysregulation (Donath and Shoelson 2011). The oral administration of Lactobacillus strains led to significant anti‐inflammatory effects, as evidenced by reduced cytokine levels, in diabetic animals, suggesting a potential biological pathway in this regard. Hopefully, the decreases in IL‐1 and TNF‐α, attributed to the mechanistic taproots, were due to probiotic colonies initiating an insulin signaling pathway approach, thereby reducing β‐cell apoptosis (Tilg and Moschen 2006). More so, a decrease in IL‐6 levels would be indicative of an improvement in the gut barrier function through decreased endotoxemia. That this occurred could be in part due to a systematic colonizing of bacterial proportions after probiotics were administered and stimulation of immune pathways of response without a marked change of systemic inflammation along with other probiotics, which in turn have lower inflammation as it relates to leading probiotics (Plaza‐Diaz et al. 2019; Zhao et al. 2018). Based on the observation of the study of prevention, it would seem that probiotics did not elicit “more” changes, and proinflammatory cytokines were changed to mild “no” provocation, so it would show that the effects of modulation would have more “effect” susceptibility alterations in increased pathological environments. The results suggest that probiotics may be beneficial as an adjuvant in therapies for modulating inflammation associated with metabolic disorders, such as T2DM. To further contextualize the anti‐inflammatory mechanisms observed, the reduction in proinflammatory cytokines may parallel environmental studies on nanoparticle bioavailability, where bioaccessible fractions dictate biological impact rather than total exposure (Pu et al. 2019). Similarly, the encapsulated probiotics in our study likely exert effects through bioavailable viable cells and metabolites (e.g., SCFAs) released in the gut, rather than total administered dose, enhancing targeted immunomodulation and minimizing systemic toxicity in diabetic models.

## 4 Conclusion

In conclusion, this study demonstrates that microencapsulated indigenous *Lactiplantibacillus pentosus* D1 and *Lactiplantibacillus plantarum* D2, isolated from traditional Iranian cheese, exhibit robust probiotic properties and significant therapeutic potential in managing type 2 diabetes mellitus (T2DM) in a streptozotocin‐induced rat model. Key findings include: (i) superior in vitro tolerance to gastrointestinal stress (survival > 64% at pH 2.5 and 0.3% bile), high cell surface hydrophobicity (> 63%), autoaggregation (> 66%), coaggregation with *E. coli* (> 51%), and broad‐spectrum antimicrobial activity (inhibition zones up to 27 mm); (ii) high encapsulation efficiency with minimal viability loss, ensuring effective intestinal delivery; (iii) significant alleviation of hyperglycemia in diabetic rats (FBG: 200.8 ± 8.4 vs. 317.1 ± 10.7 mg/dL in untreated controls, *F*(3,28) = 498.23, *p* < 0.001), improved insulin secretion (12.3 ± 1.0 vs. 9.3 ± 0.9 μIU/mL, *p* < 0.01), and better body weight retention (245 g vs. 215 g, *p* < 0.05); and (iv) marked reduction in proinflammatory cytokines (IL‐1β, IL‐6, TNF‐α; *p* < 0.001 vs. diabetic controls), indicating attenuated systemic inflammation. These effects were absent in normoglycemic animals, confirming pathology‐specific efficacy and safety (γ‐hemolysis, acceptable antibiotic susceptibility). The observed benefits likely stem from enhanced gut barrier function, SCFA production, and bioavailable probiotic fractions, paralleling bioavailability principles in environmental‐microbial interactions. Collectively, these encapsulated strains represent promising, safe, and natural adjuncts for T2DM management, warranting future clinical translation and mechanistic studies on gut microbiota modulation.

## Author Contributions

**Yousef Nami:** writing – original draft, writing – review and editing, and formal analysis. **Behnam Kafil:** methodology. **Alireza Dehnad:** supervision, conceptualization, and project administration.

## Funding

This research was supported by Razi Vaccine and Serum Research Institute under Grant [3‐35‐1851‐114‐961101].

## Conflicts of Interest

The authors declare no conflicts of interest.

## Supporting information


**Table S1:** Biochemical characteristics of 15 presumptive LAB isolates from traditional Iranian cheese.

## Data Availability

Data will be made available on request. Sequence data that support the findings of this study have been deposited in the NCBI GenBank repository with the primary accession codes PV992300 (strain D1, *Lactiplantibacillus pentosus*) and PV992304 (strain D2, *Lactiplantibacillus plantarum*).
