# Supplementary material for: Encapsulated Indigenous Lactic Acid Bacteria Strains From Traditional Iranian Cheese Alleviate Hyperglycemia and Inflammation in Streptozotocin‐Induced Diabetic Rats
Source: Food Sci Nutr. 2025 Dec 6;13(12):e71295. doi: 10.1002/fsn3.71295 (PMC12681064; doi:10.1002/fsn3.71295)
Supplement: Supplementary file 1 — Table S1: Biochemical characteristics of 15 presumptive LAB isolates from traditional Iranian cheese. [file FSN3-13-e71295-s001.docx]

Supplementary Table S1: Biochemical characteristics of 15 presumptive LAB isolates from traditional Iranian cheese.

| Isolate | Gram | Catalase | Oxidase | Citrate | Indole | Motility | H₂S | Nitrate | Arabinose | Cellobiose | Glucose | Lactose | Maltose | Mannitol | Raffinose | Ribose | Sorbitol | Sucrose | Xylose |
| --- | --- | --- | --- | --- | --- | --- | --- | --- | --- | --- | --- | --- | --- | --- | --- | --- | --- | --- | --- |
| D1 | + | – | – | – | – | – | – | – | + | + | + | + | + | + | – | + | + | + | – |
| D2 | + | – | – | – | – | – | – | – | + | + | + | + | + | + | – | + | + | + | – |
| D3–D15 | + | – | – | Variable | – | – | – | – | Variable | Variable | + | + | + | Variable | Variable | Variable | Variable | Variable | Variable |

+ = positive; – = negative. All isolates fermented glucose and were non-motile, non-H₂S producing, and catalase-negative, consistent with LAB phenotype. Only D1 and D2 showed consistent carbohydrate fermentation patterns typical of *Lactiplantibacillus pentosus* and *L. plantarum*.
